# Molecular Regulation of SASP in Cellular Senescence: Therapeutic Implications and Translational Challenges

**DOI:** 10.3390/cells14130942

**Published:** 2025-06-20

**Authors:** Hubert Klepacki, Krystyna Kowalczuk, Natalia Łepkowska, Justyna Magdalena Hermanowicz

**Affiliations:** 1 Department of Pharmacodynamics, Medical University of Bialystok, Mickiewicza 2C, 15-222 Bialystok, Poland; natalialepek2001@wp.pl (N.Ł.); justyna.hermanowicz@umb.edu.pl (J.M.H.); 2 Department of Integrated Medical Care, Medical University of Bialystok, Mickiewicza 2C, 15-222 Bialystok, Poland; krystyna.kowalczuk@umb.edu.pl

**Keywords:** senescence, senescence-associated secretory phenotype (SASP), senolytics, senomorphics

## Abstract

Cellular senescence is a complex process that significantly contributes to the pathogenesis of various diseases, including cancer and neurodegenerative disorders. It is characterized by permanent cell cycle arrest and morphological changes, such as cell enlargement and a decrease in lamin B levels. As organisms age, a secretory phenotype known as the senescence-associated secretory phenotype (SASP) develops, which produces pro-inflammatory factors that can impact surrounding tissues and promote disease. This article discusses the molecular mechanisms regulating senescence, notably the p53/p21 and p16INK4a/pRb pathways, which are crucial for inducing cell cycle arrest. While increased activity of cyclin inhibitors like p16 and p21 serves as a protective mechanism against cancer, their prolonged activation can lead to pathological effects. Additionally, the article examines therapies involving senolytics and senomorphics, which aim to eliminate senescent cells. Current research suggests that targeting senescence may represent a promising strategy for treating various diseases, improving health outcomes, and enhancing the overall quality of life as we age.

## 1. Introduction

This review provides a comprehensive and integrative overview of the complex mechanisms underlying cellular senescence, with particular emphasis on the regulation of the senescence-associated secretory phenotype (SASP) and key signaling pathways. The multifaceted nature of these processes makes them especially challenging to fully elucidate, particularly for researchers new to the field. By combining the latest molecular insights with translational perspectives and therapeutic implications, this work aims to deliver a holistic synthesis that may serve as a valuable reference for both novice and advanced researchers.

Cellular senescence is a highly dynamic, multi-step process occurring within cells. It can be triggered either by telomere shortening during each cell division (replicative aging) or by premature aging induced by cellular stress. In physiological conditions, normal cells have a limited number of divisions, and this process is associated with a gradual loss of regenerative and protective functions within the body [1]. Universal features of aging include morphological changes (cell enlargement and flattening), increased activity of senescence-associated β-galactosidase (SA-β-gal) [2], and a decrease in lamin B1 levels due to the damage to the nuclear lamina [3]. However, it is important to note that the specificity of commonly used senescence markers is limited. For example, SA-β-gal activity at pH 6.0 is not exclusive to senescent cells and can also be detected in non-senescent cells, such as neurons, macrophages, and osteoclasts, especially under stress or during differentiation [4]. The increased activity of cyclin-dependent kinase inhibitors p16INK4a and p21CIP1, which independently induce the senescence process in cells, is also a characteristic feature [5]. However, it is important to note that p16 and p21 can also be upregulated during development, tissue homeostasis, and DNA damage responses, independent of senescence. Therefore, relying on single markers may lead to false-positive identifications of senescent cells. Thus, the identification of senescent cells in vivo requires the use of multiple complementary markers and morphological features, as no single universal marker exists [6].

The resistance to apoptosis and the slowing down of proliferative processes, eventually leading to their complete inhibition, is characteristic of aging at the cellular level [7]. Numerous biochemical processes also undergo changes. There is a decrease in the synthesis of proteins essential for DNA and RNA synthesis, as well as proteins involved in glucose and fatty acid metabolism [8]. Additionally, the reduction in the production of mitochondrial proteins involved in oxidative phosphorylation [9] is an important issue that could be the direct cause of reduced energy availability in the cell, thus leading to the inhibition of cell proliferation. On the other hand, there is an increased synthesis of proteins involved in intercellular communication and membrane transport [10], adhesion molecules [11], and anti-apoptotic proteins of the BCL-2 family. A special role in maintaining cellular senescence is attributed to proteins from this group, particularly BCL-2, BCL-W, and BCL-XL, due to their strong anti-apoptotic effects [12].

## 2. Cellular Senescence: Mechanisms and Disease Implications

### 2.1. Accumulation of Senescent Cells and Their Role in Age-Related Diseases

The elimination of senescent cells decreases with age, which is likely associated with the decline in the immune function in older individuals. The loss of balance caused by the accumulation of senescent cells leads to the occurrence of diseases, including cardiovascular diseases, cancers, neurodegenerative, and degenerative diseases [13]. It is widely known that Alzheimer’s disease (AD) is a neurodegenerative disorder, with its incidence increasing with age. Studies conducted on 5XFAD transgenic mice, which exhibit multiple phenotypes associated with AD, have shown that the expression level of the *p16* gene, observed in senescent cells, is directly proportional to the level of deposited β-amyloid and inversely proportional to cognitive function [14]. Cellular senescence may also contribute to osteoarthritis. In female C57BL/6 mice, buffered saline solution (PBS) was injected into the right knees for three months, while senescent or normal fibroblasts were injected into the left knees. It was observed that the presence of senescent fibroblasts caused joint space erosion, erosion of the lateral femoral condyles, and the formation of osteophytes, which were characteristic features of osteoarthritis. Injection of normal fibroblasts also caused slight joint damage. That likely resulted from the relatively large volume of injected fluid (50 µL), as similar changes were noted in the control group [15].

### 2.2. Stress-Induced Premature Senescence (SIPS)

Premature senescence induced by stress factors [16] is another previously mentioned type of senescence. These stress factors include chemotherapeutic agents, ionising radiation, oxidative stress, and oncogenes [17]. Cytostatics, including doxorubicin, bleomycin, cisplatin, etoposide, carboplatin, and docetaxel, have the ability to induce senescence in cancer cells [18]. The strongest ability to induce cancer cell senescence is attributed to drugs that damage DNA, while drugs acting on microtubules (taxol and vincristine) have the weakest effect [19]. Studies using human breast cancer cells MDA-MB-231 and mammary gland cells MCF-10A have shown that doxorubicin, administered for 72 h at low doses (100 nM), stimulates senescence, while at higher doses (>0.1 µM), it induces apoptosis [20]. Furthermore, the analysis of biopsy material from patients with non-small cell lung cancer (NSCLC) undergoing treatment with carboplatin and taxol has shown a significant increase in the SA-β-gal expression as compared to the material from patients whose only treatment method was surgical intervention [21]. Wang Y. et al. have shown that exposure of cerebral vascular endothelial cells to acute doses of gamma radiation leads to increased production of reactive oxygen species (ROSs), resulting in premature senescence. Infrared (IR) radiation also induces senescence in those cells, with the presence of characteristic features such as SASP production, an increase in the population of SA-β-gal-positive cells, and an increase in the p16INK4a expression, which is a key factor involved in cell cycle arrest [22].

### 2.3. Molecular Triggers and Mechanisms

Oxidative stress may be induced by ROSs that include superoxide anion, hydroxyl radical, and hydrogen peroxide. The two main sources of these molecules in the human body are the mitochondrial respiratory chain and the respiratory burst of phagocytes involving NADPH oxidase [23]. To counteract their harmful effects, humans produce endogenous antioxidants, including catalases, superoxide dismutase, and glutathione derivatives. An imbalance between ROS production and antioxidant defenses leads to oxidative stress [24]. High levels of these species damage proteins and DNA at the telomeric level [25]. A subtle increase in ROSs in human fibroblasts raises the levels of senescence markers p21 and SA-β-Gal and inhibits cell proliferation [26].

Oncogene-induced premature senescence (OIS) is a potent defense against cancer, arising in response to the activation of oncogenes such as BRAF, RAS, E2F1, and AKT [27]. The best-known oncogene is RAS that is activated in response to DNA mutations [28]. An increase in its concentration induces cell cycle arrest in the G1 phase, an increase in p16 and p53 levels, and inhibition of cell division [29]. Mutations of RAS isoforms (HRAS, KRAS, and NRAS) are among the most commonly occurring oncogenes and are present in 19% of cancer patients [30]. In vitro studies have shown that RAS overexpression in breast epithelial cells induces tumor suppression and permanent cell cycle arrest [31]. Other in vitro experiments conducted on mouse lymphatic cells have shown that the NRAS overexpression triggers OIS and prevents lymphoma progression [32]. One of the most interesting facts about the anti-cancer role of OIS is the presence of the BRAF V600E mutation in human melanocytic nevi. That mutation is the most common type found in melanoma. Melanocytes with the BRAF V600E mutation, in addition to inhibited growth, exhibit characteristics of OIS, such as upregulation of p16 and positive staining for SA-β-gal. However, they retain the ability to transform into malignant tumors if BRAF-induced senescence is suppressed or reversed. Nevi are thus benign lesions that can remain stable for decades, making them one of the best available in vivo examples of OIS [33].

### 2.4. Heterogeneity of Senescent Phenotypes

Cellular senescence is a highly heterogeneous phenomenon—senescent cell phenotypes depend on the nature of the inducing stimulus, duration of stress, cell type, and tissue context. These cells exhibit variability not only in gene expression profiles and SASP components, but also in epigenetic, metabolic, and functional alterations. Such heterogeneity is evident both at the single-cell level and in whole populations, complicating unambiguous identification and classification of senescence. Understanding this diversity of senescent phenotypes is crucial for developing effective targeted therapies and reliable biomarkers of senescence in vivo [34]. Importantly, senescence is a complex cellular program accompanied by diverse phenotypic changes, including chromatin remodeling, metabolic reprogramming, and the production of a highly variable secretome. The specific composition of the SASP and other features of senescence can vary greatly depending on the cell type and the nature of the senescence-inducing factor, further underscoring the complexity and heterogeneity of this process [35]. A major obstacle in developing targeted therapies against senescent cells has been their phenotypic heterogeneity and the lack of universal biomarkers. Suda et al. demonstrated that vaccination targeting senescence-associated surface antigens enables the immune system to selectively eliminate senescent cells, which led to improvements in age-related phenotypes and extended lifespan in progeroid mice [36].

### 2.5. Immunosenescence: Aging of the Immune System and Its Consequences

Aging of the immune system (immunosenescence) affects not only adaptive cells but also leads to significant changes in the function and phenotype of NK cells. With age, there is an increase in the total number of NK cells, particularly within the CD56^dim^ population, accompanied by a decrease in the percentage of CD56^bright^ cells and a reduction in cytotoxic activity and cytokine production. These alterations contribute to the accumulation of senescent cells in the organism, chronic low-grade inflammation (inflammaging), and the development of age-related diseases. A key recent discovery is the central role of NK cells as main effectors of immunological elimination of senescent cells, both under physiological and pathological conditions. Unfortunately, the decline in NK cell activity with age leads to impaired immunosurveillance and limits the effectiveness of natural clearance of senescent cells. Understanding the mechanisms underlying NK cell dysfunction may open new therapeutic possibilities for immune rejuvenation and combating age-related diseases [37].

Furthermore, numerous studies indicate that immunosenescence is characterized by a marked decrease in the number of naive T cell subsets and the accumulation of terminally differentiated, senescent T cells. This is manifested by the loss of CD28 expression and increased levels of CD57 and KLRG1 on T cells, which are now widely regarded as key markers of the senescent phenotype. These changes are associated with reduced telomere length and impaired proliferative capacity of T lymphocytes, limiting the ability to mount effective immune responses to new antigens. Importantly, the aged immune system is marked by a shift towards pro-inflammatory cytokine profiles (e.g., increased IFN-γ and TNF-α), which further promote inflammaging and tissue dysfunction. Taken together, these findings highlight the complex interplay between innate and adaptive immune aging and underscore the importance of comprehensive phenotypic and functional characterization of both NK and T cells in the context of immunosenescence and age-related diseases [38].

Recent studies highlight that senescent T cells play a crucial role in the pathogenesis of metabolic disorders such as type 2 diabetes, obesity, and cardiovascular diseases. These cells exhibit metabolic dysregulation characterized by increased oxidative stress, mitochondrial dysfunction, and altered energy metabolism. Such changes lead to enhanced production of proinflammatory cytokines, which exacerbate chronic inflammation and promote insulin resistance. Consequently, the accumulation and impaired function of senescent T cells contribute significantly to metabolic disturbances commonly observed in the elderly population. Therefore, therapies aimed at selectively targeting or modulating these senescent cells may represent a novel therapeutic strategy to reduce chronic inflammation and improve metabolic health in aging individuals [39].

### 2.6. Dual Role of Senescence: Barrier and Risk Factor

Inhibition of cell division is a potent anti-cancer mechanism, but it is not synonymous with the loss of proliferative potential. The removal of the cell cycle block allows re-entry into the cell cycle [40]. Moreover, the preserved metabolic activity of senescent cells with persistent DNA damage is inherently associated with the SASP, the components of which include numerous pro-inflammatory cytokines and chemokines, proteases, and growth factors, which are capable of creating a tumor-promoting environment [41]. Due to its potential for both pro- and anti-cancer effects, the process of senescence is an extremely intriguing yet challenging area of research.

## 3. Selected Regulators of Senescence

### 3.1. p53/p21 Pathway: Guardian of the Genome

Inhibition of proliferation through the mechanism of senescence serves as an early blockade against passing damaged DNA to the next generation of cells [42]. The two main pathways responsible for cell cycle arrest in the G1 phase are p53/p21^CIP1^ and p16^INK4a^/pRb [43]. p53, known as the guardian of the genome, and RB (Retinoblastoma) are the most important tumor suppressor genes [44]. p53 is a protein of critical importance in maintaining genetic stability and suppressing tumor transformation. As a transcription factor, it is involved in regulating numerous cellular processes, including the cell cycle, angiogenesis, migration, DNA repair, apoptosis, autophagy, and senescence [45]. The presence of stress factors triggers the activation of kinases that phosphorylate the p53 domain [46], which, in the p53-dependent mechanism, leads to the inhibition of the cell cycle, induction of repair processes, or—in the case of failure—activation of programmed cell death. The induction of apoptosis or autophagy allows for the removal of irreversibly damaged cells [47], inter alia due to the proteins BAX, BAK, PUMA, and NOXA [48]. The p53 protein has the ability to inhibit a group of BCL-2 proteins, leading to the activation of BAX and BAD proteins and the release of cytochrome C into the cell’s cytoplasm. Cytochrome C, together with APAF-1 and caspase-9, forms the apoptosome. Subsequently, with the involvement of effector caspases, autodigestion of individual cell fragments occurs [49]. Disruption of the normal function of the p53 protein leads to loss of control over proliferation and plays a significant role in the development of many types of cancer. TP53 gene mutations are detected in approximately half of cancer cases [50]. In other cancer types, the wild-type p53 protein is observed, where overexpression of the negative regulators MDM2 and MDMX leads to its functional inactivation. The level of p53 protein in certain types of cells may have a decisive impact on the induction of apoptosis or senescence. Studies conducted on human diploid fibroblasts (IMR90) treated for 2 h with hydrogen peroxide at increasing concentrations from 50–200 µM have shown p53 expression to have been higher in apoptotic cells than in senescent cells and the control group. It may suggest that the induction of senescence or apoptosis depends on the intensity and duration of the stressor [51].

The level of the p53 protein in cells not exposed to stressors is very low, which is associated with its integration with proteins from the ubiquitin ligase group: MDM2 and MDMX. An increase in p53 protein levels within the cell induces the transcription of MDM2 and MDMX genes, resulting in the formation of the p53-MDM2/X complex, leading to degradation of p53. That type of autoregulation operates through a feedback mechanism and is responsible for controlling the level of the p53 protein in the cell. Blocking the formation of ubiquitin ligase complexes with p53 allows for the restoration and maintenance of p53-dependent signaling in cancer cells. Inhibition of MDM2 leads to cell cycle arrest and induction of apoptosis. This research is focused on compounds that simultaneously inhibit both ubiquitin ligases [52]. Potential drugs with inhibitory effects on ubiquitin-protein ligases have garnered particular interest in the potential therapy of hematological cancer, such as acute lymphoblastic leukemia or myelogenous leukemia. It is due to the relatively low frequency of p53 mutations in those types of cancer, in contrast to solid tumors, where the main cause of their formation is the mutation in the p53 gene [53].

In addition to its direct action on cancer cells, the p53 protein has the ability to activate intermediary systems that protect against carcinogenesis. One of them is the increased expression of plasminogen activator inhibitor (PAI-1) [54]. Serpina E1, the PAI-1 inhibitor, is a protein that, by inhibiting tissue plasminogen activators, acts as a regulator of the fibrinolytic system [55]. It is important to emphasize that PAI-1 is not only a key inducer of senescence but also a marker of it. Xu et al., in a study on aging endothelial cells, demonstrated a positive correlation between the level of PAI-1 and SA-β-Gal [56]. The level of PAI-1 increases with age and is involved in numerous pathophysiological processes, including metabolic syndrome [57] or chronic kidney disease [58]. The results of experiments conducted using fibroblasts have shown that the deficiency of p53 and PAI-1 leads to resistance to aging and allows for prolonged cell proliferation. The researchers have further demonstrated that in the absence of cellular p53, the overexpression of PAI-1 is sufficient to induce replicative senescence in fibroblasts [59]. Kortlever et al. have demonstrated that senescence induction through PAI-1 also occurs in response to transforming growth factor TGF-β. The role of PAI-1 in the senescence process has been documented in studies on wild-type C57Bl/6 mice that were treated with homocysteine to induce aging. It has been shown that in the experimental group, where PAI-1 inhibitors TM5441 and TM5A15 were additionally used, the level of SA-β-Gal-positive cells was significantly lower than in the control group [60]. Similar results were obtained by Ghosh et al., who observed a decrease in the number of SA-β-Gal-positive cells and a reduction in morphological changes characteristic of the aging process in cardiomyocytes, fibroblasts, and endothelial cells incubated simultaneously with doxorubicin and TM5441, compared to cells exposed to doxorubicin alone [61].

Numerous cellular processes, such as apoptosis, senescence, DNA repair, and cell cycle arrest induced by p53, occur with the involvement of PML (promyelocytic leukemia protein) [62]. The gene for promyelocytic leukemia (PML) was first discovered in the 1990s in patients with promyelocytic leukemia, where a chromosomal translocation between chromosome 15 and 17 was identified [63]. PML has the ability to induce senescence and apoptosis by stabilizing p53 [64]. It has also been shown that the binding of PML to telomerase and telomeric DNA facilitates the activation of senescence associated with telomere instability [65]. The oncogene Ras increases the expression of PML, leading to its overexpression and, consequently, p53-dependent senescence [66]. In response to ROSs, PML stimulates senescence and inhibits tumor progression by activating the gene encoding the p53 protein (TP53) and Rb. Ubiquitin-dependent enhanced degradation of PML proteins in humans is associated with tumor progression due to weakened pro-senescence activity. A deficiency of PML increases susceptibility to carcinogenic factors and oncogene-activated tumors [67]. PML deficiency is observed in numerous types of cancer, including lung, prostate, breast cancers, lymphomas, and central nervous system tumors [68].

p21 is a protein belonging to the group of cyclin-dependent kinase (CDK) inhibitors responsible for cell cycle arrest in the G1 phase. It is a transcriptional target of the TP53 tumor suppressor gene, one of the most potent genes involved in cell cycle control. In addition to p53, the transcription of p21 may be initiated by oncogenes, pro-inflammatory cytokines, and other tumor suppressors. Biochemical and genetic research indicates that p21 acts as a key effector in numerous tumor-suppressive pathways, promoting anti-proliferative actions. Moreover, by binding to the proliferating cell nuclear antigen (PCNA), p21 disrupts the PCNA-dependent activity of DNA polymerase, thereby inhibiting DNA replication and modulating various PCNA-dependent DNA repair processes. p21’s action is multifaceted, as it also participates in cell differentiation, migration, DNA repair, apoptosis, autophagy, and the process of senescence. Interestingly, recent reports suggest that under certain conditions, p21 may paradoxically promote cell proliferation and, as a result, oncogenicity [69]. It has been observed that nuclear p21 exhibits suppressive activity against tumors [70] and cytosolic p21 acts oncogenically [71]. Nuclear p21 is one of the three CDK inhibitors responsible for inhibiting cell proliferation [72]. Cytosolic p21, on the other hand, reduces the activity of capsizes, including caspases 8 and 10, pro-caspase-3, stress-activated kinase 1, as well as effectors of the apoptotic signaling pathway [73]. Induction of p21 leads to dose- and time-dependent expression of the senescence marker SA-β-gal, as well as characteristic morphological features of the aging cell [74]. The role of p21 in the induction of senescence is confirmed by studies on human glioma cell lines [75]. The overexpression of p21 induces the senescence phenotype more quickly and stably than the therapy using high-dose radiation (15 Gy), as determined by measuring the expression level of GLB1 [76].

### 3.2. p16INK4a/Rb Pathway: Cell Cycle Arrest and Tumor Suppression

**Figure 1:**
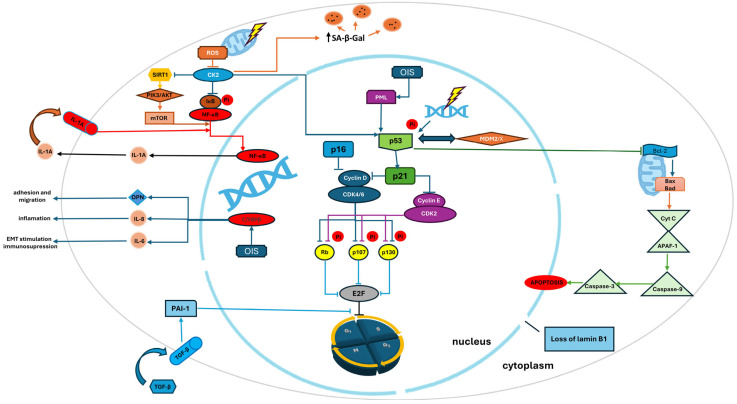
The diagram presents the key molecular pathways regulating cellular senescence, apoptosis, and the development of the SASP phenotype. The yellow lightning bolt symbolizes a damaging factor (e.g., oxidative stress, DNA damage) that initiates the cellular response. Arrows indicate activation of subsequent signaling steps, while lines ending with a blunt tip indicate inhibition of a given process or protein. The “Pi” in a circle indicates phosphorylation.

The proteins p21 and p16 regulate the level of RB phosphorylation by binding to cyclins. The Rb protein family includes Rb1, p107, and p130, which are crucial factors involved in the induction of senescence and tumor suppression. Those proteins are phosphorylated by CDKs, which reduces the ability of the Rb family members to repress the activity of the E2F transcription factor family, which is required for cell cycle progression [77]. It should be emphasized that senescent cells are characterized by the accumulation of the CDK2 inhibitor p21 ^WAF1/Cip1^ and the CDK4/6 inhibitor p16 ^INK4A^ [34]. The binding of p16 and p21 to cyclins induces hypophosphorylation of Rb, resulting in an increased ability of Rb to bind to the transcription factor E2F. The resulting RB–E2F complex inhibits the transcription of genes necessary for the transition from the G1 phase to the S phase [78]. The proteins p16 and Rb1 work together as tumor suppressors, and disruption of their function is a common cause of cancer development. It has been shown that the loss of p16 allows for unchecked cell cycle progression and bypass of OIS [79]. The increased expression of p16 may be associated not only with the induction of senescence but also with the inhibition of cell proliferation, as an increase in the p16 expression is not always linked to the appearance of SASP [80]. It has been shown that pRb, activated by p16^INK4a^, permanently and irreversibly halts the cell cycle, as the cell cycle arrest is maintained despite subsequent inactivation of Rb and p53. Such findings suggest that the p16^INK4a^/pRb pathway may activate another mechanism that plays a key role in halting cell division in the G2 or M phase. It appears that this mechanism is the inhibition of cytokinesis, due to the presence of a large number of multinucleated cells following the inactivation of pRb and p53 (Figure 1) [81]. Another study on hydrogen peroxide used on human fibroblasts and mesenchymal stem cells (MSCs) suggests that senescence is primarily maintained through the action of Rb2/p130 [82]. The same conclusions arise from studies on breast cancer cells, where doxorubicin-induced senescence is maintained through Rb2/p130 [83].

The diagram highlights two main pathways of cell cycle arrest: p53/p21 and p16INK4a/pRb, as well as mechanisms leading to apoptosis and senescence. The key pro-inflammatory proteins (C/EBP and NF-κB) are shown as the main factors sustaining SASP activation. It is worth noting that many of the pathways and associated proteins presented in the diagram are also targets of currently investigated or applied drugs, such as BCL-2 inhibitors, IL-1, IL-6, TGF-β inhibitors, MDM2/MDMX inhibitors, cyclin D/E inhibitors, and modulators of the SIRT3/PI3K/AKT pathways. Among the proteins depicted is PAI-1, for which small-molecule inhibitors have demonstrated beneficial effects in preclinical models of senescence and fibrosis. Targeting these regulatory pathways aims to modulate cellular senescence and the SASP phenotype. The diagram emphasizes the complexity of molecular regulation of senescence processes.

### 3.3. Autophagy and Mitophagy in the Regulation of Cellular Senescence

Autophagy is a fundamental, evolutionarily conserved catabolic process responsible for the degradation and recycling of cytoplasmic components, including proteins and damaged organelles, via the lysosomal pathway. Under physiological conditions, autophagy maintains cellular homeostasis and provides an adaptive response to metabolic stress or damage. Mitophagy, a selective form of autophagy, targets dysfunctional or superfluous mitochondria for degradation, thus serving as a key quality control mechanism for the mitochondrial network. Both processes are tightly regulated and are essential for the maintenance of cellular health, especially in long-lived cells. Numerous studies have demonstrated that impairment of autophagy and, in particular, mitophagy, contributes to the development of cellular senescence and aging-related phenotypes. During senescence, the efficiency of mitophagy declines, leading to the accumulation of damaged mitochondria, increased production of ROSs, and the activation of the SASP. As shown by Kelly et al. [84], suppression of basal mitophagy in primary human fibroblasts is an early and crucial event in the development of the senescent phenotype, while reactivation of this pathway through pharmacological intervention can partially reverse markers of aging and restore mitochondrial function. These findings highlight the important crosstalk between mitochondrial quality control, autophagy/mitophagy, and the regulation of cellular senescence. Similar mechanisms are also observed in AD, where disturbances in autophagy and mitophagy lead to the accumulation of damaged mitochondria and pathological proteins both in neurons and peripheral cells of patients. Defective mitophagy has been identified in studies conducted on human skin fibroblasts and in hippocampal samples derived from patients with AD. It is considered a new marker and a driving factor in the progression of this disease, and restoring its proper function may have significant therapeutic potential [85].

## 4. The Dual Role of Cellular Senescence and SASP in Tissue Homeostasis and Disease

### 4.1. The Physiological Role of SASP

**Figure 2:**
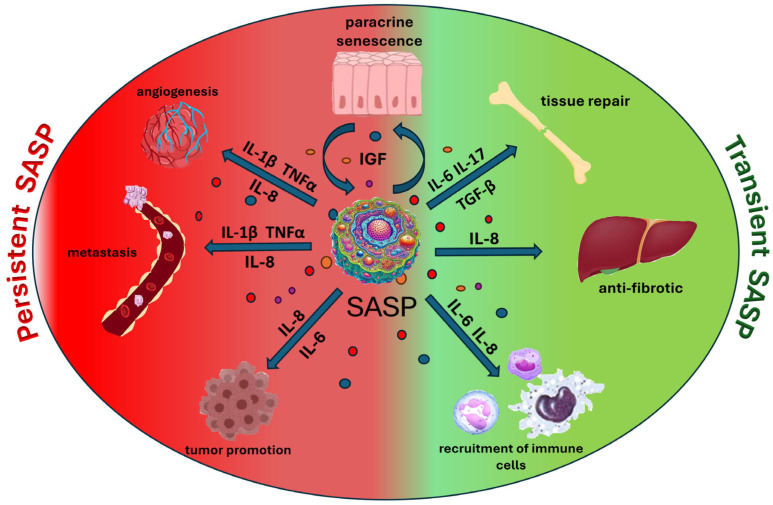
The pleiotropic functions of the SASP. The effects in the area marked in green are considered beneficial and are associated with transient SASP. The effects in the area marked in red are considered detrimental and are associated with chronic SASP.

Aging cells have the ability to produce a secretory phenotype known as SASP. As a result, numerous pro-inflammatory factors are produced and secreted, stimulating growth and modifying the extracellular matrix, which may significantly support tumor formation [86]. The presence of SASP is most commonly observed in cells with damaged DNA. Inhibition of cell division in response to other signals, such as the overexpression of p16 or p21, that are cell cycle inhibitors, does not lead to the appearance of SASP in those cells [87]. The physiological functions of SASP components, primarily IL-6 and IL-8, include stimulating immune cells such as NK cells and macrophages. Their role is to remove senescent cells from the body [88]. A prime example of that phenomenon is described in the literature regarding the regeneration of damaged liver tissue. Hepatic stellate cells, upon undergoing senescence, produce SASP, with IL-8 being the most important component protecting against fibrosis. IL-8, a ligand for the NKG2D receptor on NK cells, plays a crucial role. Cytotoxic NK cells, by destroying senescent liver cells, prevent excessive collagen production, thereby protecting against liver fibrosis [89]. The components of SASP also play a crucial role in the regeneration of damaged tissues. In bone repair, factors such as IL-6, β7 and TGF-β are involved in the process. Those molecules help modulate the inflammatory response and stimulate the repair and remodeling of bone tissue following an injury [90]. It is important to note that SASP, as a regulator of homeostasis and a factor inducing tissue regeneration, is present in young tissues and has a transient character. Chronic persistence of SASP leads to different effects and, through various mechanisms, impairs the renewal of stem cells, acts as an inducer of inflammatory states, and promotes the development of cancer [91] through enhanced proliferation and metastasis [92] or induction of angiogenesis (Figure 2) [93].

### 4.2. Main Regulatory Pathways of SASP

The mechanism of the SASP expression regulation is not fully understood; however, it is important to emphasize that not all the factors that make up SASP are regulated by the same pathways [94]. There are two main transcriptional pathways involved in the formation of SASP: C/EBPβ (CCAAT/enhancer-binding protein β) and nuclear factor kappa B (NF-κB) (Figure 1) [95]. C/EBPβ is a transcription factor playing a key role in senescence by inducing IL-6 and IL-8 in response to oncogene-induced aging, as well as regulating the levels of the pro-tumorigenic protein osteopontin (OPN) that is involved in migration, adhesion, and the induction of inflammation [96]. In studies conducted on aging fibroblasts treated with bleomycin, 127 of 834 genes responsible for synthesizing the SASP components have been discovered to be dependent on C/EBPβ [97]. Activation of NF-κB in senescent cells occurs as a result of reduced levels of protein kinase CK2 that belongs to the group of serine/threonine kinases. The decrease in CK2 levels within the cell activates the p53-p21^Cip1/WAF1^ pathway, raises the level of SA-β-gal, inactivates the deacetylase SIRT1, and activates the phosphoinositide 3-kinase (PI3K)-AKT-mammalian target of the rapamycin (mTOR) pathway [98].

Downregulation of CK2 inactivates SIRT1 and induces SASP by deacetylating the subunit of the nuclear factor NF-κB, RelA (p65) and activating protein kinase B (AKT). Inhibition of the PI3K-AKT-mTOR pathway using the mTOR inhibitor rapamycin in aging HCA2 cells reduces the NF-κB activity by approximately 80%, suggesting that this signaling pathway plays a key role in the activation of inflammation and induction of SASP. Furthermore, rapamycin is capable of inhibiting IL-1A that is cell-surface-associated and forms a positive feedback loop with NF-κB. It is important to emphasize that in senescent cells, the level of IL-1A significantly increases, thus playing a crucial role in the formation and maintenance of SASP [99].

SASP factors are secreted both paracrinally and autocrinally. Autocrine secretion contributes to reinforcing senescence within the cell, while paracrine secretion affects the surrounding healthy cells, inducing senescence in them [100]. In paracrine signaling, the insulin-like growth factor (IGF) plays a key role in influencing the microenvironment of aging cells. Studies have shown that endothelial cells and fibroblasts undergoing senescence exhibit high levels of nearly all the proteins that bind insulin-like growth factors (IGFBPs) [87]. IGFBPs constitute a group of six proteins involved in binding IGFs, thereby participating in various metabolic and regulatory processes in the human body. IGF-1 and IGF-2 are associated with the proliferation, adhesion, and migration of cancer cells, while IGF-3 and IGF-5 influence lipids that regulate cell growth [101]. It is worth noting that a retrospective study conducted on 130 breast cancer patients has demonstrated the plasma level of IGF-1 and serum IGFBP3 to correlate with recurrence frequency, tumor size, and overall survival [102]. Autocrine and paracrine secretion is regulated through positive feedback loops that ultimately strengthen the signal. If that signal becomes sustained, it leads to the development of SASP [103].

### 4.3. IL-6—Between Senescence and Cancer

The most important pro-inflammatory proteins that are part of SASP include interleukin 6 (IL-6) and 8 (IL-8), as well as TNFα [104]. Ruhland et al. [105] have demonstrated that in the tumor microenvironment of skin cancer, interleukin 6 (IL-6) has the ability to induce immunosuppression by activating bone marrow-derived suppressor cells. Elevated levels of IL-6 and consequently enhanced IL-6/JAK/STAT signaling have been observed in cancers of the breast [106], prostate [107], kidneys [108], ovaries [109] and pancreas [110]. Moreover, in the tumor microenvironment, activation of the JAK/STAT3 pathway by IL-6 occurs in both cancer cells and infiltrating immune cells, which can contribute to tumor progression [111]. In oral squamous cell carcinoma, the increase in IL-6 levels is associated with enhanced invasiveness, while suppression of IL-6 enhances the chemosensitivity and radiosensitivity of cancer cells [112]. Currently, it is widely accepted that IL-6 is a strong inducer of epithelial-mesenchymal transition (EMT). That process plays a crucial role in cancer metastasis by promoting the loss of epithelial characteristics in tumor cells and enhancing their migratory and invasive abilities [113]. It has been proven that IL-6 induces EMT in breast cancer cells. That process enhances the migratory and invasive capabilities of the tumor cells, contributing to the progression and metastasis of breast cancer [114], large intestine [115], and epithelial ovarian cancer [116]. EMT is a process that plays important roles in wound healing, fibrosis, and cancer development. In that process, epithelial cells lose their epithelial characteristics and transform into mesenchymal cells. However, it is a reversible and highly dynamic process [117]. Cells undergoing the described transitions can exist in an intermediate state, which facilitates their survival, colonization of other organs, and metastasis due to reduced cell adhesion [118]. Recent data show that IL-6, beyond its well-known role as a secreted SASP factor, also acts inside the producing cell to maintain the senescent state through an intracrine mechanism. In a rat pituitary tumor model (MtT/S cell line), knockout of IL-6 led to loss of senescence and the acquisition of a highly tumorigenic phenotype in vivo. Restoration of IL-6 expression in these cells reinstated senescence and blocked tumor growth in NOD/SCID mice. This mechanism involves intracellular IL-6 interacting with IL-6R, cytosolic DNA, and activating the cGAS-STING-NFκB pathway. These findings suggest that intracellular IL-6 is essential for maintaining the senescence barrier and preventing malignant transformation, at least in the pituitary context [119].

### 4.4. IL-8 and TNFα in Cancer Progression and Inflammation

A strong pro-inflammatory factor included in SASP is also IL-8, a chemokine produced by macrophages and macrocytes, that has the ability to activate neutrophils. It has been proven that elevated levels of IL-8 are present in numerous cancers and contribute to the development of many of their characteristic features, such as neoangiogenesis, increased proliferation, and metastasis. It has also been shown that there is a correlation between the IL-8 expression and the ability to form metastases [120]. Clinical research results indicate that the level of IL-8 is significantly higher in areas where metastases are formed as compared to primary sites [121]. The fact that IL-6 and IL-8 act together is also significant. They play an important role in the development of precancerous conditions as well as tumor progression by enabling the invasion of epithelial cells into the basement membrane [122]. TNFα is another component of SASP, playing a significant role in maintaining the inflammatory state. Its presence is often observed in the inflammatory environment of many cancers, and its chronic presence is associated with a strong pro-tumorigenic effect [123]. In A549 lung cancer cells, TNFα enhances the EMT induced by TGFβ [124]. In breast cancer, a synergistic effect of TNFα and IL-1β has been observed, stimulating angiogenesis and remodeling of the extracellular matrix. The levels of both molecules are strongly expressed in cancer cells and are correlated with the stage of the disease and the risk of cancer recurrence [125]. A summary of the key SASP components—including cytokines, chemokines, growth factors, and their biological functions—is presented in Table 1.

### 4.5. Pathological Microbiota and the Induction of Cellular Senescence and SASP

The gut microbiota, often referred to as a “hidden endocrine organ”, plays a key role in maintaining host homeostasis by regulating metabolism, immunity, and aging processes. Increasing evidence suggests that disturbances in the composition and activity of the microbiota accelerate cardiovascular aging, particularly through the induction of SASP in endothelial cells. The accumulation of endothelial cells exhibiting SASP leads to impaired vascular function, chronic inflammation, and an increased risk of age-related cardiovascular diseases. Recent data from in vivo studies in mice show that the microbial metabolite PAA (phenylacetic acid)—produced predominantly by pathogenic strains such as *Clostridium* sp. ASF356—initiates chronic oxidative stress in endothelial cells by enhancing mitochondrial H_2_O_2_ production, which results in DNA damage, SASP activation, and persistent cellular senescence. At the same time, aging is associated with a marked decline in protective short-chain fatty acids, especially acetate, which physiologically suppresses SASP and supports redox homeostasis. As a result, the combination of excessive PAA, originating from pathological *Clostridium* species, and a deficiency of SCFAs accelerates the accumulation of senescent endothelial cells and exacerbates vascular dysfunction typical of aging [162].

Notably, similar microbiota-driven mechanisms have also been implicated in the pathogenesis of obesity-associated liver cancer. In vivo mouse studies have demonstrated that a high-fat diet (HFD) and obesity lead to a marked expansion of Gram-positive bacteria from the phylum Firmicutes, which are typically part of the physiological gut microbiota. Under these conditions, the excessive growth of Firmicutes results in increased production and hepatic translocation of specific microbial metabolites—particularly deoxycholic acid (DCA) and lipoteichoic acid (LTA). These metabolites reach the liver, where they induce senescence and SASP in hepatic stellate cells. Through TLR2 signaling, LTA stimulates the expression of COX2 and the production of immunosuppressive prostaglandin E2 (PGE2) in these senescent cells. Excess PGE2 dampens antitumor immune responses by modulating T cell activity and promoting an immunosuppressive microenvironment, thereby facilitating tumor progression. Importantly, this mechanism—characterized by the accumulation of senescent cells with an active SASP—was also observed in human liver cancer associated with non-alcoholic steatohepatitis (NASH), underscoring the clinical relevance of the gut–liver axis and the broader impact of microbial metabolites on tissue senescence and disease development [163].

These findings underscore the key role of gut microbiota–derived metabolites in driving cellular senescence and SASP across different tissues, linking microbial dysbiosis to chronic inflammation, vascular and hepatic aging, and the progression of age-related diseases. Although most current evidence comes from in vivo studies in mice, similar mechanisms are increasingly being observed in human tissues, highlighting the translational potential of targeting gut microbiota to prevent or mitigate age-associated pathologies.

## 5. Pharmacological Strategies Targeting Senescent Cells: Senolytics, Senomorphics, and Therapeutic Challenges

**Figure 3:**
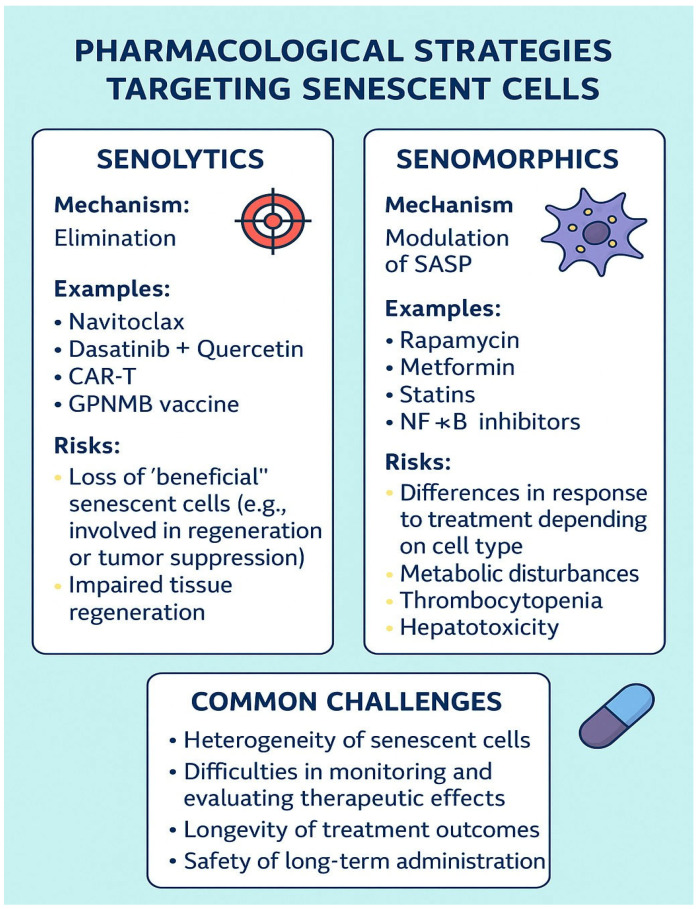
Overview of pharmacological strategies targeting senescent cells: senolytics, senomorphics, and common therapeutic challenges.

Senolytics and senomorphics represent two principal pharmacological strategies aimed at targeting senescent cells in the context of aging and age-related diseases. The main differences, examples, mechanisms of action, and risks associated with these therapeutic approaches, as well as the most important challenges in their application, are summarized in Figure 3.

### 5.1. Senolytics

Senescent cells are usually removed from the body within a few days or weeks, primarily by NK cells [164]. Disruption of the effective removal of those cells is explained by the threshold theory of senescent cell accumulation. It assumes that surpassing a certain accumulation threshold leads to a dominance of the paracrine effect of SASP over the body’s elimination capacity. As a result of that phenomenon, senescence, tissue destruction, and suppressive effects on the immune system cells occur [165]. The consequence of the immune system suppression is the inhibition of apoptosis and the elimination of senescent cells from the body, as well as the high expression of anti-apoptotic pathways (SCAP) [166]. It has been shown that elevated levels of anti-apoptotic proteins from the BCL family are often observed in senescent cells [167] as well as in cancer cells, which allows them to survive and may be a cause of resistance to the applied therapy [168]. Yosef R. et al. [169], using human IMR-90 fibroblasts, mouse embryonic fibroblasts (MEFs), as well as double-transgenic K5-rtTA/tet-p-14 mice induced for senescence, have demonstrated that simultaneous inhibition of BCL-W and BCL-XL by siRNA or ABT-373 (a BCL protein inhibitor) significantly enhances apoptosis in senescent cells. Those studies have also proven that administration of the ABT-373 inhibitor to mice effectively eliminates both ionizing radiation-induced senescent cells in the lungs and those induced by p14ARF-dependent activation of p53 in epidermal senescent cells. The possibility of pharmacologically eliminating aging cells may pave the way for new therapeutic strategies for age-related diseases.

Classical small-molecule senolytics, although effective, often act in a non-selective manner and may also affect healthy cells. Recent developments in senolytic therapies have focused on enhancing selectivity and reducing off-target effects through the use of advanced modalities, such as targeted CAR T cells and senolytic vaccines. These approaches are designed to specifically recognize and eliminate senescent cells by exploiting unique surface markers or molecular signatures, thereby improving both safety and efficacy compared to classical small-molecule senolytics. Notably, while these innovative strategies offer significant improvements in selectivity, certain risks remain, including the possibility of cytokine release syndrome or off-target toxicity, particularly if the targeted markers are not entirely specific to senescent cells. Moreover, most current evidence is based on preclinical models, and further clinical studies are necessary to fully assess their safety and therapeutic potential in humans [170].

### 5.2. Senomorphics

As summarized in Table 2, both first-generation, non-selective senolytics and second-generation, more targeted agents are included, illustrating the evolution and diversity of current strategies being explored for the selective elimination of senescent cells.

Senomorphic agents represent a class of pharmacological compounds that, rather than eliminating senescent cells, aim to modulate their function and mitigate their deleterious effects on the tissue microenvironment. Substances with senomorphic activity include rapamycin, metformin, statins, resveratrol, aspirin, NF-κB pathway inhibitors (e.g., SR12343), p38MAPK inhibitors (SB203580, UR13756, BIRB796), JAK/STAT inhibitors (ruxolitinib), ATM inhibitors (KU-55933, KU-60019), as well as natural polyphenols such as apigenin, kaempferol, genistein, epigallocatechin gallate (EGCG), oleuropein, and hydroxytyrosol [188]. However, there are obvious issues, such as the translational gap between results obtained from animal models (especially mice) and humans, difficulties in adjusting the therapeutic dose, and the occurrence of adverse effects. Moreover, as the authors of the publication emphasize, much of the data on the efficacy and mechanisms of action of senomorphics comes mainly from preclinical studies, while clinical trials in humans are still scarce and at an early stage. Another challenge is the heterogeneity of senescent cells and differences in response to treatment depending on cell type, tissue, and organism. Some senomorphics may cause side effects such as metabolic disturbances, thrombocytopenia, hepatotoxicity, or impaired wound healing (e.g., rapamycin), which further limits the widespread use of these substances in human therapy. Therefore, as the authors point out, further large-scale clinical studies are necessary to assess the efficacy, safety, and long-term effects of senomorphic use in humans [189].

Table 3 includes a selection of senomorphics that are either currently in clinical research or have already been investigated in completed clinical trials.

### 5.3. Therapeutic Challenges

Research on senolytics and senomorphics is currently not only justified but indeed of utmost importance from the perspective of public health and translational medicine. Convincing evidence comes from large population studies, such as the work by Schafer et al., which demonstrated that circulating concentrations of SASP factors are strongly associated both with chronological age and with so-called biological age, reflecting the degree of accumulated damage and health deficits in an individual. Moreover, elevated levels of selected SASP proteins (including GDF15, FAS, OPN, TNFR1, ACTIVIN A, CCL3, IL-15) allow for better prediction of clinical complications in older adults than age alone or traditional risk factors [199]. These human population data are further supported by recent systematic reviews and meta-analyses in animal models, which have demonstrated that pharmacological interventions targeting senescent cells—such as senolytics and senomorphics—can effectively reduce senescent cell burden, improve organ function, and alleviate age-related disease phenotypes [200,201]. Furthermore, a recent meta-analysis of proteomic datasets of the senescence-associated secretory phenotype (SASP) identified both core and context-specific secreted factors, highlighting common pathways as well as stage-dependent pro-inflammatory mediators. These results underscore the complexity and dynamic heterogeneity of SASP, providing valuable insights for the development of targeted senotherapeutic strategies [202].

Despite the promising results of senolytic therapies in preclinical and early clinical studies, important limitations remain. Senolytic drugs can lack complete selectivity, risking the removal not only of deleterious, SASP-secreting senescent cells but also beneficial senescent cells involved in tissue repair, regeneration, or cancer suppression. Additionally, the long-term effects of depleting senescent cells in humans are unknown and may include impaired wound healing, immune dysfunction, or even increased risk of tumorigenesis, underscoring the need for further well-controlled clinical trials before broad application of senolytics can be recommended [203]. In addition to these concerns regarding selectivity and potential adverse effects, another key challenge is the issue of treatment timing and long-term safety. The risk of initiating therapy at an inappropriate time, as well as uncertainty regarding the long-term effects and safety of senolytic treatment, represent significant challenges. The optimal timing for the start of therapy remains unknown—too-early elimination of senescent cells may impair wound healing or immune responses, whereas interventions introduced too late may not be able to reverse established, irreversible tissue damage. Furthermore, there is a lack of long-term studies in animal models and humans evaluating the side effects of chronic senolytic administration, including the risk of disrupting tissue homeostasis or the emergence of new, unexpected pathologies.

## 6. Conclusions

In summary, the main highlights of this review are as follows:

- Cellular senescence acts as both a barrier to cancer and a driver of chronic diseases via the SASP.
- Senolytic and senomorphic therapies represent promising approaches, but their selectivity, clinical efficacy, and safety require further study.
- Most clinical data are still preliminary; preclinical findings should be cautiously interpreted before translation into clinical practice.
- The heterogeneity of senescent cells and lack of specific markers remain key challenges.
- Circulating SASP factors, including IL-6, may serve as valuable clinical biomarkers for predicting health status and complications in the elderly.

## Figures and Tables

**Table 1:** Senescence-associated secretory phenotype components and their functions. TNFα-tumor necrosis factor alpha, Groα-Growth-regulated oncogene alpha, Groβ-Growth-regulated oncogene beta, Groγ-Growth-regulated oncogene gamma, MCP-1-Monocyte chemoattractant protein-1, CXCL-11-C-X-C motif chemokine ligand 11, MIC-1-Macrophage inhibitory cytokine-1, MMP-1-Matrix Metalloproteinase-1, NAMPT-Nicotinamide phosphoribosyltransferase, STC1-Stanniocalcin-1.

Components of SASP	Factor	Effects	Reference
Cytokines	IL-1α	Formation and maintenance of SASP	[126]
		Promote cancer progression	[127]
	IL-1β	Induce angiogenesis	[114]
		Promote tumor invasiveness	[128]
		Induce immunosuppression	[129]
		Increase vascular permeability	[130]
	IL-6	Induce EMT	[106]
		Inhibition of dendritic cells (DCs)	[131]
		Induce cell proliferation	[132]
	TNFα	Remodeling of the extracellular matrix	[114]
		Reinforce cellular senescence	[133]
Chemokines	IL-8	Promote tumor growth and migration	[134]
		Induce cellular senescence	[135]
	Groα	Induce EMT	[136]
		Promote metastasis	[137]
	Groβ	Promote cell proliferation	[138]
		Promote recruitment and polarization of M2 macrophages	
	Groγ	Inhibition of tumor cell apoptosis	[139]
		Mediates cancer cells migration and proliferation	[140]
	MCP-1	Promote tumor growth and migration	[141,142]
		Induction of angiogenesis	[143]
		Activation of tumor-associated macrophages	
	CXCL-11	Induce tumor-suppressive cells (NKs, CTLs)	[144]
		Promote cancer cells migration and proliferation	[145]
Growth factors	IGFBP3	Induce premature cellular senescence	[146]
	IGFBP7	Induce cellular senescence	[147]
	TGF-β	Promote tumor cells apoptosis	[148]
		Induce EMT and metastasis	[149]
	MIC-1	Promote cancer cells invasiveness	[150]
		Acceleration tumor cells proliferation and invasion	[151]
Others	PAI-1	Synergistic TGF-β induced cellular senescence	[152]
		Inhibition of tumor cells apoptosis	[153]
		Promote polarization of M2 macrophages	[154]
	MMP-1	Promote cancer cells migration and proliferation	[155]
		Induce angiogenesis	[156]
		Promote cancer cells invasiveness	[157]
	NAMPT	Promote polarization of M1 macrophages	[158]
		Induce inflammation	[159]
	STC1	Inhibition of tumor migration and invasion	[160]
		Promote tumorigenesis	[161]

**Table 2:** Selected senolytic compounds, their mechanisms of action, experimental models, clinical status, main findings, and reported adverse effects. AML—acute myeloid leukemia, CAR T cells—chimeric antigen receptor T cells, CRh—complete remission with partial hematologic recovery, CRi—complete remission with incomplete hematologic recovery, CRS—cytokine release syndrome CXCL—C-X-C motif chemokine ligand, gen-generation (e.g., first-generation or second-generation senolytics), GM-CSF—granulocyte-macrophage colony-stimulating factor, HSCs—hepatic stellate cells, HUVECs—human umbilical vein endothelial cells, IL—interleukin, MCP-1—monocyte chemoattractant protein-1, MEFs—mouse embryonic fibroblasts, MIC-1—macrophage inhibitory cytokine-1, MMP—matrix metalloproteinase, mTOR—mechanistic target of rapamycin, NAMPT—nicotinamide phosphoribosyltransferase, NF-κB—nuclear factor kappa B, NOXA—pro-apoptotic Bcl-2 family member, Nrf2—nuclear factor erythroid 2-related factor 2, OXR1—oxidation resistance protein 1, PBMCs—peripheral blood mononuclear cells, PI3K—phosphoinositide 3-kinase, PPARγ—peroxisome proliferator-activated receptor gamma, uPAR—urokinase-type plasminogen activator receptor, VEGF—vascular endothelial growth factor.

Compound/Class	Mechanism of Action	Cell Type/Model	Clinical Status	Gen.	Main Findings/Conclusions	Adverse Effects/Risks (Reported)	Ref.
Navitoclax (ABT-263/737)	BCL-2 family inhibition	IMR-90 (in vitro) MEFs (in vitro) HUVECs (in vitro) mouse lung tissue (in vivo) mouse skin (in vivo) irradiated mice (in vivo)	No clinical trials	I	Effectively clears senescent cells, particularly after irradiation or stress-induced senescence; promotes apoptosis by inhibiting BCL-2	thrombocytopenia	[169,171,172]
Navitoclax + Ruxolitinib	BCL-2 inhibition (navitoclax), selective JAK1/JAK2 inhibition (ruxolitinib)	Human myelofibrosis patients (in vivo)	Yes, Phase II NCT03222609	I	Improved spleen volume, reduced symptoms, and bone marrow fibrosis in myelofibrosis patients	Thrombocytopenia (88%), anemia, fatigue	[173]
Navitoclax + Venetoclax + Decitabine	BCL-2 family inhibition (navitoclax), selective BCL-2 inhibition (venetoclax), DNA hypomethylation (decitabine)	Human refractory acute myeloid leukemia (AML) patients (in vivo)	Yes, Phase Ib NCT05222984	I	20% achieved complete remission with incomplete/partial hematologic recovery (CRi/CRh), 60% had reduction in bone marrow blasts, 20% proceeded to allogeneic stem cell transplantation	Thrombocytopenia, neutropenia, anemia, febrile neutropenia, gastrointestinal symptoms (nausea, diarrhea)	[174]
Venetoclax (ABT-199)	Selective BCL-2 inhibition	Human sarcoma cell lines STS93, STS109, STS117 (in vitro)	No clinical trials	I	Induces apoptosis in irradiated, senescent sarcoma cells.	Not applicable	[175]
Quercetin	BCL-2/Bcl-xL inhibition Axl/STAT3/IL-6 pathway suppression, EMT blockade	Jurkat T cells (in vitro) U87MG, U373MG, glioblastoma cells (in vitro) glioblastoma PANC-1 (in vitro) PATU-8988 pancreatic cancer cells (in vitro)	No clinical trials	I	Induces apoptosis in cancer cells, suppresses EMT and invasiveness, reduces STAT3/IL-6 signaling	Not applicable	[176,177,178]
Dasatinib + Quercetin (D + Q)	Multi-kinase inhibition (dasatinib) BCL-2/Bcl-xL inhibition (quercetin)	Postmenopausal women aged 55–80 with osteopenia or generally healthy (in vivo)	Yes, Phase II NCT04313634	I	In exploratory analyses, women with higher baseline levels of T cell senescence markers showed improvements in bone formation, reduced bone resorption, and increased radial bone mineral density after treatment	No significant adverse effects reported	[179]
		Adults with diabetic kidney disease (in vivo)	Yes, Phase II NCT02848131	I	Short-term D + Q treatment reduced the burden of senescent cells (p16^INK4A^, p21^CIP1^, and SA-β-gal positive cells) in adipose tissue and skin, decreased SASP factors and inflammatory markers	Mild to moderate gastrointestinal symptoms, transient chills, or headache observed.	[180]
Piperlongumine	OXR1 protein degradation	Human peripheral blood mononuclear cells (PBMCs) (in vitro)	No clinical trials	I	Senolytic compounds reduced the epigenetic age of blood samples in vitro; supports rejuvenation potential of senolytic treatment in human cells	Not applicable	[181]
Curcumin	Nrf2 and NF-κB inhibition	Human intervertebral disc cells (in vitro)	No clinical trials	I	Induces apoptosis in senescent cells	Not applicable	[182]
	PPARγ/p53 activation	Rat hepatic stellate cells (in vivo)	No clinical trials	I	Induction of senescence in rat HSCs; increased expression of p16 and p21	No significant adverse effects reported	[183]
Ouabain	Na^+^/K^+^-ATPase inhibition	Senescent IMR-90 human fibroblasts (in vitro)	No clinical trials	I	Selectively induces apoptosis in senescent cells by upregulating NOXA; a broad-spectrum senolytic effect has been demonstrated both in vitro and in vivo	Potential cardiac toxicity	[184]
Fisetin	PI3K/Akt and mTOR pathway inhibition	MRL/lpr mice with lupus nephritis (in vivo)	No clinical trials	I	Reduced senescent tubular epithelial cells, inhibited fibroblast proliferation, reduced fibrosis, and improved kidney function	No significant adverse effects reported	[185]
		Postmenopausal women, survivors of stage I–III breast cancer after chemotherapy (in vivo)	Yes, Phase II NCT05595499	I	Study ongoing; results not yet available	Study ongoing; adverse effects not yet reported	[186]
mGL392 (micelle-encapsulated GL392)	Selective delivery to senescent cells via lipofuscin-binding domain dasatinib-induced apoptosis	Senescent IMR-90 human fibroblasts (in vitro)	No clinical trials	II	Effectively eliminates senescent cells, demonstrated improved tissue function and reduced SASP in animal models.	No significant adverse effects reported	[187]
uPAR-targeting CAR T cells	uPAR-targeted recognition and elimination of senescent cells (CAR T cells)	Senescent IMR-90 (in vitro) mouse models of liver fibrosis (in vivo) mouse models of lung adenocarcinoma (in vivo)	No clinical trials	II	Selectively eliminate senescent cells, restore tissue homeostasis, reduce fibrosis, and improve physical function in vivo	CRS, transient weight loss, hypothermia, increased serum cytokines at high doses; mild, transient macrophage infiltration in lungs; no significant toxicity at therapeutic doses	[170]
GPNMB-targeted senolytic vaccine	Induction of immune responses against GPNMB-expressing senescent cells	Senescent mouse fibroblasts (in vitro) aged mouse models (in vivo)	No clinical trials	II	Selectively eliminates senescent cells, improves physical function, delays age-related pathologies, and extends lifespan in mice	No significant adverse effects reported	[36]

**Table 3:** Selected senomorphic agents investigated in preclinical and clinical studies. The table summarizes their mechanisms of action, experimental models or patient populations, clinical status (including ongoing and completed trials), main findings, and reported adverse effects or risks. ADCS-ADL—Alzheimer’s Disease Cooperative Study—Activities of Daily Living (a scale assessing daily functioning in Alzheimer’s patients), Aβ—amyloid beta, AMPK—AMP-activated protein kinase, APP/PS1—transgenic mice carrying genes associated with Alzheimer’s disease (Amyloid Precursor Protein/Presenilin 1), CSF—cerebrospinal fluid, DMARD—Disease-Modifying Antirheumatic Drug, FGF-2—Fibroblast Growth Factor 2, HUVEC—Human Umbilical Vein Endothelial Cells, IMR-90—human lung fibroblast cell line, MDC—Macrophage-derived chemokine, MMP9—Matrix Metalloproteinase 9, MMSE—Mini-Mental State Examination, STAT1/3—Signal Transducer and Activator of Transcription 1/3, TLR4—Toll-Like Receptor 4.

Compound/Class	Mechanism of Action	Cell Type/Model	Clinical Status	Main Findings/Conclusions	Adverse Effects/Risks (Reported)	Ref.
Rapamycin (Sirolimus)	mTOR inhibition suppression of SASP factors	Genetically heterogeneous mice (in vivo)	Approved for other indications (immunosuppression), not approved for anti-aging	Feeding rapamycin late in life (600 days of age) significantly extended median and maximal lifespan in both male and female mice. Effect observed despite late intervention.	No significant adverse effects reported	[190]
		Older adults with periodontal disease	Yes, Phase II—RAPID	Results pending	No significant adverse effects reported	[191]
		Women aged 35–45 (in vivo)	Yes, Phase I NCT05836025	Ovarian aging deceleration (~20%) potential menopause delay (~5 years) Improvement of memory, energy, skin, hair	No significant adverse effects reported	[192]
Metformin	Upregulation of endoplasmic reticulum (GPX7), reduction of cellular oxidative stress	IMR-90 human lung fibroblasts (in vitro)	Approved for other indications (type 2 diabetes), not approved for anti-aging	Alleviation of cellular aging, reduction of senescence-associated markers, improvement of redox homeostasis	Not applicable	[193]
	AMPK activation, improved insulin sensitivity, possible reduction of neuroinflammation	Older adults with amnestic Mild Cognitive Impairment (in vivo)	Yes, Phase II NCT00620191	Improvement in memory and cognitive function in metformin group versus placebo	No significant adverse effects reported	[194]
Simvastatin	Induction of endothelial nitric oxide synthase (eNOS) via Akt pathway, inhibition of endothelial senescence	HUVEC—human endothelial cells (in vitro) Mice (in vivo)	Approved for hypercholesterolemia, no clinical trials for anti-aging in this model	Suppression of endothelial cell senescence, improved endothelial function, reduction of senescence markers both in vitro and in vivo	No significant adverse effects reported	[195]
	Reduction of cholesterol levels, potential modulation of neuroinflammation	Older adults with mild to moderate Alzheimer’s disease (in vivo)	Yes, Phase III NCT00053599	No slowing of cognitive decline or disease progression despite significant lipid lowering	No significant difference in serious adverse effects compared to placebo	[196]
Resveratrol	Inhibits TLR4 oligomerization and pro-inflammatory signaling (NF-κB, STAT1/3, Akt), suppresses cytokine (IL-6, TNF-α, IL-1β) production; inhibits Aβ-induced microglial/macrophage activation	Murine microglial and macrophage cell line (in vitro)	No clinical trials	Significantly decreased cytokine production and inflammatory signaling in LPS- and Aβ-stimulated cells.	Not applicable	[197]
	Reduces number of activated microglia around amyloid plaques in the brain (partly independent of amyloid burden)	APP/PS1 transgenic mice (in vivo)	No clinical trials	Reduces activation of microglia surrounding amyloid plaques in the cortex	No significant adverse effects reported	
	Reduction of neuroinflammation (↓ MMP9 in CSF), induction of adaptive immunity (↑ IL-4, MDC, FGF-2 in CSF	Patients with mild to moderate Alzheimer’s disease (in vivo)	Yes, Phase II NCT01504854	52 weeks of treatment significantly reduced MMP9 in CSF, increased anti-inflammatory and neuroprotective cytokines (IL-4, MDC, FGF-2) in CSF, attenuated the decline in cognitive (MMSE) and functional (ADCS-ADL) scores compared to placebo	Well-tolerated overall; most common: nausea, diarrhea, weight loss	[198]

## Data Availability

No new data were created or analyzed in this study. Data sharing is not applicable to this article.
